# Resource footprints and their ecosystem consequences

**DOI:** 10.1038/srep40743

**Published:** 2017-01-23

**Authors:** Francesca Verones, Daniel Moran, Konstantin Stadler, Keiichiro Kanemoto, Richard Wood

**Affiliations:** 1 Industrial Ecology Programme, NTNU, Trondheim, Norway; 2 Faculty of Economics and Law, Shinshu University, Matsumoto, Japan

## Abstract

A meaningful environmental impact analysis should go beyond the accounting of pressures from resource use and actually assess how resource demand affects ecosystems. The various currently available footprints of nations report the environmental pressures e.g. water use or pollutant emissions, driven by consumption. However, there have been limited attempts to assess the environmental *consequences* of these pressures. Ultimately, consequences, not pressures, should guide environmental policymaking. The newly released LC-Impact method demonstrates progress on the path to providing this missing link. Here we present “ecosystem impact footprints” in terms of the consequences for biodiversity and assess the differences in impact footprint results from MRIO-based pressure footprints. The new perspective reveals major changes in the relative contribution of nations to global footprints. Wealthy countries have high pressure footprints in lower-income countries but their impact footprints often have their origin in higher-income countries. This shift in perspective provides a different insight on where to focus policy responses to preserve biodiversity.

Environmental footprints look at the effect that a population’s consumption or lifestyle has on the environment. Traditional footprints, based on a consumption-based accounting principle^1^, report on resource use and emissions, i.e. on environmental pressures. In the language of the DPSIR framework (drivers, pressure, state, impact and response)^2^, used by e.g. the EU Environment Agency and the OECD, traditional footprints link drivers and pressures (Fig. 1). In studies of environmental footprints however, there have so far been limited attempts to continue along the lines of the DPSIR framework and link pressures to the state of the environment in order to assess the environmental *consequences* of consumption. Policy should respond to actual environmental impacts, not simply pressures. For example, water use policy should consider the impacts caused by water consumption, not just the volume of consumption itself^3^.

There are many studies of environmental footprints, including those that consider carbon^4^, raw materials^5^ and water^6^ which report on the resource demands associated with consumption^4^,^7^,^8^. These footprint studies focus on one specific pressure only, e.g. emissions of greenhouse gases (GHGs), tonnage of primary materials used, or cubic meters of water utilized. One challenge of reporting impact footprints has been how to combine different resource footprints, which are using different metrics and units. The methodology of the Ecological Footprint handled this by expressing all pressures in terms of land area affected^9^,^10^,^11^,^12^, though this approach has received some criticism^13^,^14^,^15^,^16^. One recent approach of interest is that by Lenzen *et al*.^17^, where the authors linked drivers (consumption drivers on economic production) directly to state (number of affected species). However, that study did not connect drivers to impacts. Moreover, their approach was retrospective, i.e. it linked already existing threat levels to current trade flows and did not follow the environmental causality chain^18^.

Our aim is therefore to contribute towards a more complete representation of the global consequences of anthropogenic consumption and production on biodiversity by combining pressure footprints with their ecosystem impacts. Traditional pressure footprints follow multiple trade and transformation steps along global supply chains to connect consumer purchases to the primary resources required to provide them by using multiregional input-output (MRIO) models. In order to continue along the DPSIR chain from pressures to impacts, we need to link environmental consequences to these pressures. Environmental impact analysis is concerned with how a resource demand or emission (e.g. a litre of surface water consumed) creates damages via multiple impact pathways (e.g. wetlands habitat loss^19^, malnutrition potential^20^, etc.). One promising tool for this linking is life cycle impact assessment (LCIA). LCIA attributes a damage to every emission or resource use in order to indicate the consequences of anthropogenic actions. In recent years, there has been substantial development in LCIA both in terms of impact categories considered and in spatial detail. By combining the Eora MRIO global supply chain database^21^ and the LC-Impact LCIA model^22^ we can quantify the consequences of anthropogenic actions and obtain a comprehensive picture of how consumer demand is linked to actual environmental impacts via production and resource use. The result is a new “impact footprint” extended to estimate the actual environmental impact – not just pressure – of consumer demand. For this impact footprint we consider the damage on species richness (as proxy for biodiversity) for the following impact categories: climate change, terrestrial acidification, marine and freshwater eutrophication, land stress and water stress^22^. This damage is measured in units of ‘potentially disappeared fraction’ (described below)^23^.

In measuring the impact on biodiversity it is important to consider multiple stresses together since species extinctions are rarely (though occasionally^24^) caused by a single stressor^25^. In this study, we consider environmental consequences of multiple pressures, through multiple, independent impact pathways, to arrive at total impact. LC-Impact provides spatially differentiated factors for all impact categories on a global level, which we link to spatially explicit production, and on to the MRIO model, allowing the identification of location-specific impacts. This spatial detail is highly relevant for impacts related to biodiversity due to the large differences in both species richness and species vulnerability across spatial scales.

Our approach provides a more comprehensive representation of the consequences of anthropogenic impacts on species richness from trade. Our measure of biodiversity impact combines species richness with species vulnerability information (see methods). This provides an alternative, arguably better, account of impact on biodiversity than existing metrics such as resource demand (classic footprints), species richness^21^,^26^, land occupation^27^, or human appropriation of net primary productivity^28^. Our objectives are to investigate which countries drive the biggest impacts on biodiversity through which impact pathways and to investigate the relationship between pressure and impact from the perspective of consumption and trade drivers. We hypothesize that this type of footprint will show different distributions of impacts between countries than traditional footprints.

## Results

### Total ecosystem impact footprints

The ecosystem impact footprints reveal how much of the impacts caused by consumption in one country damage ecosystems in other countries. We present results that show both pressure footprints and associated biodiversity loss using the measure of “potentially disappeared fractions of species” (PDF). This is a measure that traditionally takes species richness into account, but is extended in LC-Impact to also account for species vulnerability. The PDF metric reflects the potential extinction of species, within a specific time associated with resource uses or emissions, which are leading to habitat losses or degradation. We followed thirteen different types of pressures through eight distinct impact pathways (climate change, marine and freshwater eutrophication, terrestrial acidification, water and three types of land use), affecting three different types of ecosystems (aquatic, terrestrial, marine) (see Methods for further details). Depending on the pressure considered, a sharp difference is observed between environmental pressure footprints and the new impact footprints. Here we highlight the most striking results. Further information is provided in the online Supporting Information (SI).

**Figure 2:**
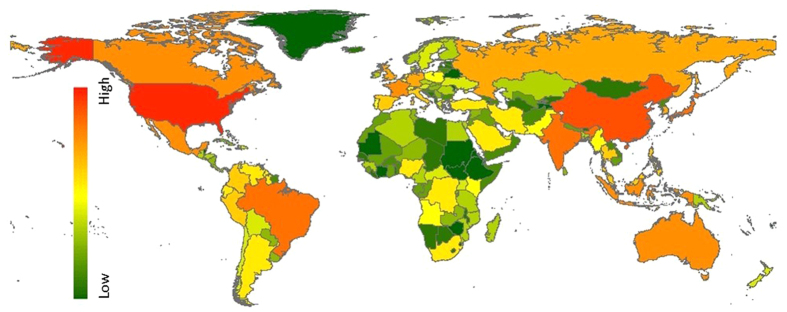
The ecosystem impact footprint of nations, summed over all impact pathways (unit PDF, year = 2012). Map created with ArcMap 10.2(ww.esri.com/software/arcgis)^68^.

Consumers in the US and China have the largest total ecosystem footprint (Fig. 2), followed by Brazil, India, and Japan. These countries have high overall consumption, high resource footprints, and also high numbers of endemic species domestically. While Brazil and India have lower per-capita consumption than major EU economies, these countries have large populations and much of the consumption of these countries is for domestic resources. The pressures from this domestic use of resources has higher biodiversity impacts than for imported products (see SI, compare land shares of impact versus pressure). Impacts from land occupation, water stress and expected climate change impacts from CO_2_ emissions, constitute the three most important contributions to the overall impacts, accounting for more than 99% of our modelled impacts. However, the individual contributions of the different impact pathways vary per country. In some arid countries, such as Australia, Iran and Pakistan, water consumption impacts are larger than land occupation impacts, while in others (e.g. US or China) the reverse can be observed.

**Figure 3:**
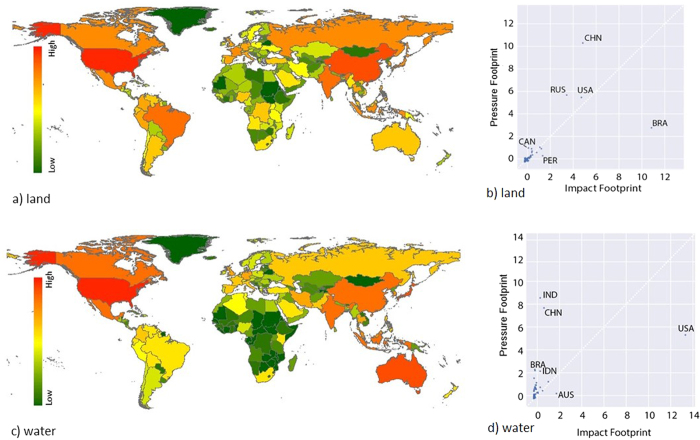
Impact footprints of nations are shown for total land occupation (**a**), and water consumption (**c**). The ranking of nations based on their footprint differs sharply when viewed as traditional pressure footprints (y-axis) vs. impact footprints (x-axis): (**b**) land stress (**d**) water stress (both axis standardized (see methods), dotted line representing similar pressure and impact footprints). Correlations and maps for all pressures assessed are provided in the SI (Figure S2–S10), as well as a double logarithmic depiction of the pressure vs impact relationships. Maps created with ArcMap 10.2(ww.esri.com/software/arcgis)^68^

Applying the same pressure on environments with different resource availability and / or species richness will lead to different impacts at the national level (see Fig. 3). For land and water use, the difference of having an impact footprint or a resource footprint are evident (Fig. 3b,d; if the impact and resource footprints were the same, all countries would fall along the 45° line, as is the case for CO_2_ emissions – see Figure S2 in the SI).

### Impacts related to land occupation

For land occupation, the largest differences between pressure and impact footprints are found for Brazil, China, and Russia. The difference observed in Brazil is again due to the large share of domestic production in the Brazilian footprint, (90.2%, see SI), as well as the large number of (often endemic) species in Brazil. About 9.5% of the worlds species are estimated to exist in Brazil^29^, and according to IUCN, there are 5249 red-listed animal species present in Brazil. Almost one fifth of these red-listed species (966) are endemic^30^,^31^, which due to their often small habitat areas have higher vulnerability scores^19^,^22^ (see also methods). These endemic species thus have a higher weight than non-endemic species in the PDF metric. We see this also in the bilateral results (Table 1 and SI): land-intensive goods from Brazil create a higher impact footprint than comparable goods from other countries. In contrast, some other countries, such as China and Russia, have lower than average ecosystem consequences of land use. This is due to relatively low impact factors at the national average^22^ (three to five times smaller for China than for Brazil), attributable to the smaller species numbers (1958 and 4835 red-listed animal species in Russia and China, respectively)^31^, and endemics (22 animal species in Russia and 525 in China)^30^. For an individual analysis of the damage factors see references^19^,^32^. Many countries contribute to large land pressure footprints in Russia and China. However, this is not true for the land-based impact footprints. When looking at the contribution of China to global trade, 11.5% of total land pressure embodied in trade comes from China (see SI,), yet only 3.7% of total biodiversity impact embodied in trade comes from China (see SI - Total_ImpactFootprint_and_shares.xlsx, column GM). For the US (which hosts 6738 red-listed animal species, 366 endemic animals)^30^,^31^, resource and impact footprints have almost the same relevance. This is because of their higher-than-average domestic impacts (see Table 2), and strong trade links to e.g. Canada, and China which show lower-than-average ecosystem consequences due to limited numbers of endemic and vulnerable species (only 0.3% of Canada’s animal species are endemic).

The origin of imported goods is central for determining the impact footprint of countries because of the spatial difference in impact factors. Countries with strong trade links to areas of high species richness and high endemism (reflected in the vulnerability score) will ultimately affect these hotspots of biodiversity (e.g. the US importing from Brazil). Impact footprints are hence relatively higher than pressure footprints for those countries with either high impact factors in domestic or imported goods. Whilst on the other hand, results for countries that shift from high pressure footprints to lower ecosystem impact footprint (e.g. Russia Fig. 3b) exhibit either a) low domestic ecosystem impact factors or b) a lack of strong trade links with the biodiversity rich regions in South America and South East Asia, as is the case for Russia, for example.

### Impacts related to water consumption

For water consumption, the differences between pressure and impact footprints are even more pronounced (Fig. 2d). Primarily the USA and Australia, but also Japan and Indonesia, have a larger share of global impact footprint due to water consumption than global water consumption footprint, while for e.g. India and China, the reverse can be observed. These six countries account for 43% of global water consumption (pressure). However, the same countries account for 75% of the global water impact footprint. One of the most striking differences is found for Australia, which accounts for 0.7% of the global water pressure footprint but 8% of the water impact footprint. Australia has an exceptionally high level of species endemism (e.g. 92% of native vascular plants and 94% of native amphibians)^33^. Every lost species in Australia has a high impact on the global species count, and therefore LC-Impact assigns a large weight to Australian species. This, together with the high water consumption of the Australian economy^1^ and because 70% of water footprint is domestic (SI), explains the high impact of water use for Australia. The US (whose main water trade partner is Canada, see Table 1) is similar, with the domestic share of the resource footprint (65%) being smaller than the domestic share of the impact footprint (97%) (SI).

The highest ecosystem consequences of US water consumption footprint occur domestically (see Table 1 and SI). This is because water consumption in the US has a higher biodiversity impact than in trading partner nations. The US, in addition to harbouring over 6700 species (of which more than 360 are endemic)^30^, contains extensive wetland and waterbody areas. The latter constitutes the main habitat for a large number of vulnerable wetland species.

In other major economies, we find that due to the dominance of Australia and the USA in potential global aquatic biodiversity loss, the relative importance of domestic water consumption is reduced. For example, China’s water pressure footprint is mostly due to domestic consumption (81%), but in impact terms, domestic water impacts are only a striking 2.5% of the total Chinese water footprint. Instead, 31% of China’s water impact footprint occurs in Australia, and 52% occurs in the USA.

### Impacts from greenhouse gas emissions

Since the damages from most GHG emissions are the same regardless of the point of emission, LC-Impact provides global values for damages from GHGs (exceptions to this, which include volatile compounds with spatially explicit impacts for climate change, and CO_2_ from bioenergy due to temporal and albedo effects^34^, are not considered in current LC-Impact metrics, and have a non-zero, but minor contribution to global GHG emissions). These values for different GHGs are time-integrated, and therefore they essentially scale all the emissions from the consumption-based accounting in the same way. Thus, resource footprints and impact footprints correlate perfectly for greenhouse gases.

### Shift in location of impacts

The country level results show a strong trend: because of the generally higher endemism and species richness in high-income countries there is a significant shift in location of impacts from low-income countries to higher income countries when switching from a pressure to impact based metric when calculating footprints.

To test this finding we aggregate results to the World Bank classification of income status. Results clearly show a marked shift from low, lower-middle and upper-middle to high income status (Table 2). Such changes occur for all spatially explicit impact factors (see SI). For example we see that 57% of the resource footprint of high income nations falls on other high income nations, but after considering impact, 94% of the total impact footprint falls on other high-income nations (upper left cell in Table 2). Upper middle- and lower middle-income countries also exert a higher impact on high income nations than the simple resource footprint indicates (3% vs 26% and 3% vs 11%). This finding is explained by the occurrence of higher species richness, higher species endemism, and higher species vulnerability in high-income countries.

A full set of maps across key consumer regions and indicators is provided in the SI, all confirming the findings presented in Fig. 3 and Table 1: different distributions of impacts are revealed when the footprint is calculated in terms of ecological impact instead of resource use.

## Discussion and Conclusion

### Shift in locations of impacts depending on proxies

Common environmental pressure indicators used in consumption-based accounting are not necessarily good proxies for ecosystem impacts. Ecosystem impact footprints provide a novel perspective to assess the role of different countries driving global species loss. Traditional resource footprints have generally shown that high-income countries displace their environmental burdens onto middle and lower-income countries^15^. While resource footprints do suggest that environmental burden-shifting occurs, we see the impact footprint is actually suffered more acutely in higher-income nations after considering species vulnerability.

We found a substantial shift from lower income countries to more affluent countries that carry the burden of biodiversity impacts (Table 2).

For pressure footprints, the contribution of relatively lower income countries is central to the carbon, land and water footprints of relatively higher income countries. In contrast, the analysis of the ecosystem impact footprints shows that affluent countries become more relevant. China is a major source of environmental degradation embodied in trade when pressure footprints are considered. However, in terms of impact footprints this is no longer the case, as impacts are lower in China due to species richness and endemism inherent to the different impact pathways. This finding argues against the hypothesis of ecologically unequal exchange, which posits that environmental burdens are typically shifted from higher-income countries to lower-income ones. While ecologically unequal exchange has been explored well by theorists^35^,^36^, empirical support for the hypothesis is mixed^15^,^37^,^38^. However, as further discussed below, also the metric developed here has its limitations and uncertainties.

### Major contributors to ecosystem impacts

Our results show that land use is the pressure with the dominating contribution to the impact footprint with 66% of PDF impact being caused by land occupation. This is consistent with a finding from IUCN that habitat loss/degradation is by far the dominant threat to biodiversity^39^. Our results further show that water stress is an additional highly important impact pathway. Wetlands and rivers are biodiversity-rich regions which have already lost more than 50% of their original coverage and are deteriorating faster than any other ecosystem^40^,^41^. Consequently, the high number of PDF from water stress obtained from the impact assessment model and the combined MRIO-LC-Impact model indicate that damage to wetlands has major biodiversity impacts. Furthermore, biodiversity rich countries often exhibit a high level of endemism, which is reflected in a high impact factor for those countries. This, in turn, can result in a high impact footprint when domestic water consumption is also high, such as the case for Australia and the USA.

### The need for spatially refined assessments

Ecosystem deterioration due to land use is highly site specific and calls for spatially differentiated assessments. Due to the large difference in species richness and vulnerability, the land area occupied for production is not a suitable proxy for associated biodiversity impacts in trade, as shown by Chaudhary and Kastner^27^. This is in line with Myers *et al*.^42^, who globally identified 25 hotspots that need to be prioritized for conservation. Hotspots were defined as areas with both exceptionally high endemism and habitat loss. Two of those hotspots alone are found in Brazil, and one each in China, Peru and the US. One of them, the Atlantic forest of Brazil, has been reduced in its original area by more than 90%, while still containing more than 2% of both the world’s endemic plant and vertebrate species^42^. This large habitat loss combined with a high level of endemism (and therefore high vulnerability in LC-Impact) increases the impacts of land occupation, a fact clearly visible in the results for Brazil. In comparison, China, although home to another highly threatened biodiversity hotspot (only 8% of the original area remaining)^42^, generally shows lower impact footprint as compared to the resource footprint. This is due to smaller fraction of the country area that is covered by the biodiversity hotspot and the comparably lower number of endemism found (1.2% and 0.7% endemic plant and vertebrate species, respectively). In general, aggregating the impacts and factors on a country level, i.e. aggregating biodiversity-rich regions with larger regions of lower biodiversity, will decrease the importance of the whole country. An area of convergence between LCIA models (which work at subnational spatial scales such as ecoregions) and MRIO models (which operate mostly at a resolution of nations) is therefore the geographical resolution of the link. The issue is most pertinent for large countries with varied landscapes and resource availability, such as China, the USA, Brazil, etc. In these countries species richness, endemism and the scarcity of available resources may vary over several orders of magnitude. Aggregating impacts on a country level therefore blurs the results. While it is possible to deal with such a level of spatial detail in LCIA, this is traditionally not the case for MRIOs, since trade flows are usually available on a country-basis only.

Accurate linking of spatial detail is crucial, as argued by Godar *et al*.^43^. Irrigation areas, as another example, may lie in areas with little impact on ecosystems and could therefore be unproblematic. It would therefore also here be beneficial to conduct analyses at small spatial scales, if the spatial resolution of Eora would allow for this. However, we checked the alignment between water use data and impact factors data for the examples of maize, wheat and cotton in the US (see SI) and found that water is indeed to a large extent consumed in areas with higher ecosystem impact, thus being to a certain degree aligned.

Data on production and environmental pressures are nowadays generally available at the grid-cell level. Therefore, the basic MRIO framework is amenable to this enhancement. Subnational areas, or grid cells, may potentially be treated as additional regions. Developing combined sub-national / multi-national MRIO accounts is an active field of work^43^,^44^,^45^. While this improved detail would increase the accuracy of the results, we have no reason to expect *a priori* that this shortcoming introduces any systematic bias into the results. The issue of error in MRIO models has been discussed extensively in the literature^46^,^47^,^48^ especially in regards to aggregation and allocation error.

### Limitations

A major advance of LC-Impact is the inclusion of spatial detail and the consideration of per-species vulnerability (see Methods). Having impact factors available at a fine spatial detail is a significant step forwards as this allows for calculating differentiated impacts for different regions. Moreover, differences in species richness and the vulnerability of species can be taken into account. There is, however, a potential information bias regarding the presence of threatened species, even for well-studied taxonomic groups including mammals^49^, meaning that it may appear there are more species threats in one region simply because there is more threat reporting in that region. The IUCN Red List data used in this study may be biased because data availability varies between regions and taxonomic groups towards (1) better-studied taxonomic groups and (2) countries which study taxonomic groups more intensively, i.e. mostly western countries^50^, which may contribute to the shown shift in results from lower-income to higher-income countries. Nevertheless, LC-Impact is based on the latest available data of multiples species of several taxonomic groups (mammals, birds, amphibians, reptiles). This ensures an adequate representation of the overall species present. In addition to biased over-reporting, under-reporting may also occur. Several taxa, in particular plants, insects and fungi, are still underrepresented in the IUCN dataset. The issue of sample bias is discussed by Larsen *et al*.^51^. Given this potential for sample bias from the IUCN Red List data, the LC-Impact results should be regarded as a “best case” scenario. There may be additional species, and/or species with unknown extents of occurrence, which are omitted from this study. We therefore most likely underestimate the level of biodiversity threat.

We combine the impacts of several pressures into one final result (see Table 3). This allows us to see a more complete picture of the set of impacts that occur alongside each other instead of just focusing on single pressures. However, despite the common unit used, we have to stress that we do not assess the potentially synergistic effects of cumulative impacts (such as changes in the local water availability due to a change in land use and thus a change in water storage capacity in the ground). These complex interactions are so far missing in LCIA and will need to be addressed in the future. Constant improvements of both LC-Impact and MRIO models are also required in terms of coverage. Eutrophication impacts are comparatively very small, for example, which is most likely an underestimation. Eora provides data for agricultural sources of available nitrogen and phosphorus (see Table 3), but not for other sources, such as sewage. This means that we neglect part of the nutrient sources responsible for eutrophication. On the other hand, characterization factors for freshwater eutrophication only cover phosphorus and the one for marine eutrophication only nitrogen since each is the major limiting nutrient in the respective ecosystem. However, impacts from an over-availability of the other nutrient (e.g. nitrogen in freshwater systems) do have an influence regarding eutrophication as well, which is currently neglected.

### The need for covering the consequences of anthropogenic activities

Current biodiversity loss and ecosystem degradation puts our planet beyond its boundaries^52^. This has been acknowledged by the global community and several international initiatives have been set in place. The Aichi Biodiversity Targets of the Convention on Biological Diversity aims to significantly reduce habitat loss and degradation by 2020^53^ and the European Commission announced the goal to halt biodiversity loss within the EU by 2020, while also increasing their contribution to prevent and reduce global biodiversity loss^54^. Most recently, two of the newly released sustainable development goals of the United Nations directly address the issue of biodiversity loss (SDG 15) and ecosystem degradation (SDG 14). These initiatives must be accompanied with the appropriate tools to assess the drivers behind biodiversity loss and to design responses to counter it. Until now, resource usage was used as a proxy for the impact. However, our study highlights the need for going further along the DPSIR framework in order to cover the consequences of anthropogenic trade and consumption and not just the pressure of those activities. This allows one to highlight the trade flows, resource use and emissions with the most impact and helps consumers to identify trade-offs between different trade options and their consequences. The proposed framework provides a possibility to track the impact of biodiversity loss through all stages of the DPSIR framework and identify the underlying drivers. This enables the design of targeted policy responses to halt further ecosystem degradation.

## Materials and Methods

### Overview

In this study a standard Leontief demand-pull model^48^ was applied to an environmentally extended multi-region input output (EE-MRIO) table in order to calculate a consumption-based account, the footprints of resource demand. The resource demands are the non-monetary inputs to production (land, fertilizer, phosphorus (P) and nitrogen (N) emissions, etc., for a full list of considered pressures see Table 3). These pressures were translated into measures of environmental impact using the characterization factors from the LC-Impact model^22^. Those factors report the impact per unit of resource demand, e.g. per kg of P emissions into freshwater systems. The used unit is globally potentially disappeared fraction of species (PDF), i.e. the potential global extinction of species. Each impact pathway acts independently from the other ones, not considering synergistic effects. It is common in LCA to sum impacts within one area of protection (i.e. ecosystem quality, human health or natural resources). Since all impact categories converging into one area of protection have one common unit, comparison across the categories is facilitated. The pressures for calculating the impacts, considered in the study are provided in the Supporting Information. The characterization factors can be download from the LC-Impact webpage (we used the version available in April 2016). The pressures and corresponding characterization factors are summarized here in Table 3. We used the EE MRIO database Eora as basis for the calculations^21^, with added data for phosphorus and nitrogen^55^,^56^,^57^,^58^,^59^.

### LC-Impact

LCA consists of four phases (goal and scope definition, life cycle inventory, life cycle impact assessment and interpretation)^60^,^61^. During the life cycle inventory, emissions and resource uses that are relevant for the life cycle of a product or a process are collected, whilst life cycle impact assessment assesses the impact of the emissions and resource use.

LC-Impact^22^ is a spatially-differentiated life cycle impact assessment (LCIA) method. It covers 11 main impact categories with up to four sub-categories. Factors for assigning impacts (characterization factors) are available for three overarching categories (human health, ecosystem quality and resources). Only those impact categories leading to damages on ecosystem quality are relevant here. The factors used are shown in Table 3 and ecosystem types covered by the different impact categories are shown in Table 4. For land occupation six broad categories exist (annual crops, permanent crops, pasture, urban, extensive and intensive forestry)^32^. Here we use the “annual crops” factor for agricultural production, “pasture” for pasture land and a weighted average of intensive and extensive forestry for the pressure “forested area”. Every country has its own set of characterization factors for these six land use types. Within one country, the factor we used here is however an average value and thus does not change (all pasture land in China gets the same CF, for example). Additional information to the background of each impact category can be found in the SI and in Verones *et al*.^22^.

The advances in LC-Impact over existing LCIA methods are: 1) spatial differentiation, 2) consolidation of ecosystem impacts and 3) coverage of new and improved impact pathways.

#### Spatial differentiation

Most ecosystem impacts, such as impacts from water consumption, vary hugely between regions, not only because of the different distribution of resources, but also because of differences in species richness (see below). Global average characterization factors are therefore either severely under- or overestimating the impact. Therefore, except for climate change, all impact categories used are spatially differentiated. The resolution differs between the different impact categories, due to differences in the relevance of spatial detail: while terrestrial ecoregions offer sufficient spatial detail for assessing the impacts of land use on terrestrial ecosystems, they are unsuited for freshwater-related impacts, where watersheds were considered to be more appropriate. An important trade-off in the choice of the spatial scale in each impact category was data availability, which dictated the possible spatial detail. For use with MRIO analysis, we aggregated the characterization factors per country based on the spatial distribution of the volume of the corresponding emissions (see *Eq3* below).

#### Consolidation of ecosystem impacts

Contrary to other LCIA approaches LC-Impact accounts for global species losses. In different impact categories, we have different taxa or groups of taxa that act as proxies for biodiversity (see ref. 22 and SI). By taking the global species numbers of those taxa into account, we can calculate the impacts on a global level (global extinctions)^22^. Moreover, since different species have different degrees of vulnerability to further changes in their habitat, we introduced a vulnerability score in water and land use (the most detailed impact categories), accounting for the fact that some species might be widespread and robust towards change and others may be at risk of extinction^32^,^62^. Vulnerability scores include information on the geographical ranges and the IUCN threat level of each species. The geographic range area reflects the intrinsic rarity of a species (pointing towards endemism), whilst the threat level indicates already occurring stress due to existing threats. The threat categories (least concern, near-threatened, vulnerable, endangered and critically endangered) were translated into a linear scale from 0.2 to 1. The range of the vulnerability scores extends from zero to 1 (amphibians), 0.04 (birds), 0.1 (mammals) and 0.2 (reptiles). Reasons for these large differences are the larger number of species in higher threat categories for amphibians than for others, as well as an on average one order of magnitude smaller geographical range area^63^.

For impact categories where information on several taxa was available individually, we calculated taxon-specific characterization factors, before harmonizing them^63^. We only considered species in our datasets that are not yet extinct, therefore we are not accounting for extinctions that already took place. However, we do account for potential future global extinctions in all impact categories, thus harmonizing the potential loss indicator, which has often been a mix of local and global extinctions in older LCIA methods.

#### Coverage of impact pathways

We used all currently available LC-Impact impact categories that are relevant for ecosystem quality and matched them to the available MRIO data regarding climate change, freshwater and marine eutrophication, terrestrial acidification, water consumption and several types of land occupation (see Table 3). While climate change was already well-developed in earlier LCIA methods, the rest of the impact categories have experienced a large shift of improvement. It is for the first time now, that water consumption impacts on biodiversity (terrestrial and aquatic) are included on an endpoint level. The same is true for marine eutrophication. Models for land use, terrestrial acidification and freshwater eutrophication have been significantly improved from previous approaches, incorporating more data and spatial detail to the calculation of the factors. For an overview of relevant background publications for the LC-Impact chapters, see SI, section 1.

#### Resource impact and pressure comparison

The impacts and pressures of resource usage are measured in different units. In order to compare them, we independently standardized each pressure and impact footprint value *x* by calculating standard scores *x*^*s*^(Equation 1):


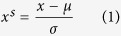


with *μ* being the mean of the corresponding pressure or impact footprint vector and *σ* its standard deviation. The result of this transformation is a standardized ranking of nations based on their pressure or impact footprint. Since we focus on the most important countries regarding biodiversity impacts, we provide these results directly in the graphs (Fig. 3b/d and SI Figs S2–S11). However, due to the large number or relatively small countries clustering in these graphs we also provide a depiction of log transformed rankings in the SI (SI Figs S11–S21).

### EE-MRIO

Here we briefly outline the basic consumption-based accounting model and discuss how the MRIO environmental pressure accounts were linked to spatially-explicit characterization factors. We start from the calculation of consumption based accounts, and refer the reader to literature on the basics of environmentally extended input-output analysis^64^.

In environmentally-extended MRIO analysis there is a *r* × *s* environmental satellite account *F* which records the total inputs of each non-monetary input *r*_*i*_ into sector*s*_*j*_. The inputs in *r* may be traditional resources such as labor, or capital, or, in our case, environmental pressures such as land area, and pollution such as phosphorus (P) and nitrate (N) runoff (see Table 3 for the considered pressures in this study). In this way pollution is accounted as an input to production. The MRIO databases include satellite accounts for a range of resources or emissions, and in the Leontief demand pull model are normalized by units of sector output *x*_*j*_. The resource footprint is calculated as


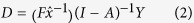


where ^ denotes diagonalization, *D* is the total environmental footprint associated with the consumption of goods and services *Y* (here of individual nations); 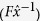 is the normalized environmental satellite described above, showing the environmental inputs per unit of monetary output of a sector; (*I* − *A*)^−1^ is the “total requirements matrix”, based on the inverse of the identity matrix (showing a unit output of each sector), minus the inputs to each sector shown in *A*. The *A* matrix can be considered the “technology matrix”, showing the intermediate (from the economy) inputs per unit output. Inverting the matrix subtraction captures the full supply-chain or “life-cycle” impacts of production per unit of consumption. The steps described so far are the basis of standard EE-MRIO analysis^65^,^66^,^67^.

From the LC-Impact model we obtain a matrix of characterization factors *C* in units of impact (for this study, PDF), per kg, ha, etc., of stress exerted at high spatial resolution. LC-Impact developed impact characterization factors for several pressure substances e.g. phosphorus emissions, CO_2_ emissions, and so on. Some of the pressures, e.g. CO_2_, have the same characterization factor no matter where the substance is emitted, but most others have different characteristics depending on where the stress was exerted/emitted. For these latter ones, LC-Impact provides spatially-differentiated results (resolution shown in Table 3). As discussed in the limitations discussion, the MRIO pressure accounts are generally not spatially differentiated – often, they are simply national totals – so it was necessary to calculate an average characterization factor for each pressure at the country level. This was done by calculating the emission-weighted (or resource-weighted for water and land use) average characterization factor for each impact category in each country. With a raster map *F*^*rm*^ of pressure emissions of type *r* in grid cell *m* and identically sized raster map *C*^*rm*^ of characterization factors for that pressure (recalling that *C* may consist of entirely uniform values in the case of impacts that are not spatially differentiated, e.g. global warming), both containing values only within the borders of a particular country (denoted by subset 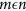), the average characterization factor for that pressure in that country is


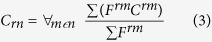


The resultant *C* has dimensions *r* × *n* but since it is only the countries, not the sectors within them, that are spatially distinguished we then repeat the columns of *C* and expand it to size *r* × *s* so it can be multiplied against 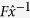.

We may now re-calculate the original footprint *Q* in terms of impact associated with consumption by pre-multiplying the system with the impact-characterized pressure intensity using a Hadamard product (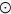):


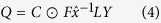


Now our impact footprint *Q* has the same numerator units of *C*.

Finally, to investigate where a footprint originates we may select any pressure *r* and elucidate the sector-country pairs where that stress was originally exerted in service of satisfying the demand bundle *Y* by diagonalising the source of the pressure/impact (signified with a the diagonal hat) :


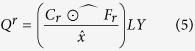


This variant *Q*^*r*^ shows disaggregated sources of each impact, from which we aggregate sectors to give a *n* × *n* table for each impact *r* in PDF. Rows indicate the country-sector pair where an impact is felt and the columns indicate the country whose consumption drives that impact. An equivalent derivation of source/destination of pressures can also be applied to *Eq2.*

## Additional Information

**How to cite this article**: Verones, F. *et al*. Resource footprints and their ecosystem consequences. *Sci. Rep.* **7**, 40743; doi: 10.1038/srep40743 (2017).

**Publisher's note:** Springer Nature remains neutral with regard to jurisdictional claims in published maps and institutional affiliations.

## Supplementary Material

Supplementary Information

Supplementary Dataset 1

Supplementary Dataset 2

Supplementary Dataset 3

Supplementary Dataset 4

Supplementary Dataset 5

Supplementary Dataset 6

Supplementary Dataset 7

Supplementary Dataset 8

Supplementary Dataset 9

Supplementary Dataset 10

Supplementary Dataset 11

Supplementary Dataset 12

Supplementary Dataset 13

Supplementary Dataset 14

Supplementary Dataset 15

Supplementary Dataset 16

Supplementary Dataset 17

Supplementary Dataset 18

Supplementary Dataset 19

Supplementary Dataset 20

Supplementary Dataset 21

Supplementary Dataset 22

## Figures and Tables

**Figure 1:**
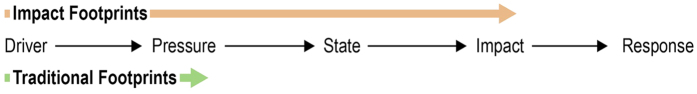
In the DPSIR framework, traditional resource footprints consider only drivers and environmental pressures, following the production and trade networks linking them. Since environmental policy should respond to impacts, not pressures, impact footprints are proposed as a better method for connecting drivers (consumption) all the way to their environmental consequences.

**Table 1:** Comparison between main contributors to pressure footprints (including impact in the consumer country) and impact footprints of USA, India, Japan, UK and China, respectively.

Consumer	Resource	Greatest in	But Impact Footprint greatest in
	Footprint		
USA	Water Demand	Self (65%), Canada (4%)	Self (71%), Japan (1%), UK (1%), Israel (1%)
	Land Area	Self (75%), Canada (8%)	
	CO_2_	Self (74%), China (8%)	
India	Water Demand	Self (91%)	Self (63%), Australia (4%), Singapore (2%), UK (2%)
	Land Area	Self (88%)	
	CO_2_	Self (86%), China (3%)	
Japan	Water Demand	Self (14%), Ethiopia (10%), USA (9%), China (9%)	Self (57%), USA (7%), Australia (6%), Thailand (1%)
	Land Area	Self (22%), China (15%), USA (14%), Canada (8%)	
	CO_2_	Self (59%), China (16%), USA (3%)	
UK	Water Demand	Self (16%), Nigeria (10%), India (6%), France (5%)	Self (64%), USA (2%), Ireland (2%), France (1%)
	Land Area	Self (49%), Ireland (12%), France (6%), Germany (3%)	
	CO_2_	Self (44%), China (13%), USA (5%), Russia (3%)	
China	Water Demand	Self (80%)	Self (40%), USA (5%), Japan (5%), Australia (4%)
	Land Area	Self (91%)	
	CO_2_	Self (91%)	

Land area comprises cropland, pasture, and forest area. CO_2_ encompasses fossil CO_2_ and CO_2_ from biomass burning.

**Table 2:**
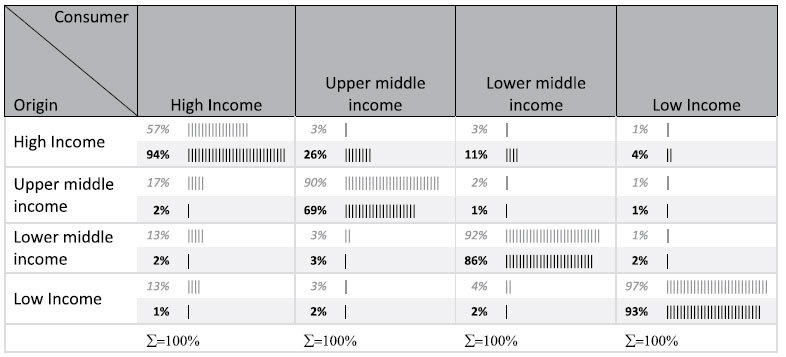
Embodied land footprint (cropland as example) transfers between income groups (origin on rows, consumers on columns) change when viewed as traditional pressure footprints (unshaded italicized rows) and as impact footprints (shaded, bold rows).

**Table 3:** Summary of considered pressures from the MRIO and the corresponding impact categories from LC-Impact.

Aggregated impact	Pressure	Impact pathway
Climate change	CO_2_ (Gg)	Climate change [PDF·yr/kg] (global)
	Biomass Burning (Gg CO_2_)	Climate change [PDF·yr/kg] (global)
	GHG-CH_4_ (Gg)	Climate change [PDF·yr/kg] (global)
	GHG-N_2_O (Gg)	Climate change [PDF·yr/kg] (global)
Water use	Blue water consumption (m^3^)	Water stress [PDF·yr/m^3^] (0.05° × 0.05°)
Land occupation	Agricultural Land area (Ha; itemized by 172 crops)	Land occupation, annual crops [PDF·yr/km^2^] (ER)
	Pasture (Ha)	Land occupation, pasture [PDF·yr/km^2^] (ER)
	Forested area (Ha)	Land occupation, forestry [PDF·yr/km^2^] (ER)
Eutrophication	Agricultural phosphorous application by fertilizer and manure (kg)	Freshwater eutrophication [PDF·yr/kg] (0.5° × 0.5°)
	Agricultural nitrogen application by fertilizer and manure (kg)	Marine eutrophication [PDF·yr/kg] (LME)
Terrestrial acidification	NH_3_ (Gg)	Terrestrial acidification [PDF·yr/kg] (2° × 2.5°)
	NO× (Gg)	Terrestrial acidification [PDF·yr/kg] (2° × 2.5°)
	SO_2_ (Gg)	Terrestrial acidification [PDF·yr/kg] (2° × 2.5°)

In brackets the unit and the spatial resolution of the used characterization factors is given. LME stands for large marine ecoregion, of which 64 exist in the coastal waters on the globe. ER stands for terrestrial ecoregion (825 around the world).

**Table 4:** Relationship between impact categories and covered ecosystem types.

Impact category	Ecosystem covered
Climate change	Terrestrial
Freshwater eutrophication	Aquatic
Marine eutrophication	Marine
Water consumption	Aquatic and terrestrial
Land occupation	Terrestrial
Terrestrial acidification	Terrestrial
