# Supplementary material for: Resource footprints and their ecosystem consequences
Source: Sci Rep. 2017 Jan 23;7:40743. doi: 10.1038/srep40743 (PMC5256307; doi:10.1038/srep40743)
Supplement: Supplementary Information [file srep40743-s1.pdf]

Resource footprints and their ecosystem consequences

Francesca Verones[1]*, Daniel Moran[1], Konstantin Stadler[1], Keiichiro Kanemoto[2], Richard Wood[1]

[1]Industrial Ecology Programme, NTNU, Trondheim, Norway
[2]Institute of Decision Science for a Sustainable Society, Kyushu University, Fukuoka, Japan
*corresponding author: francesca.verones@ntnu.no

Supporting Information

1. LC-Impact: Origin of the factors and further discussion

LC-Impact was originally a research project that was funded by the European Union under the seventh framework programme, with the aim to establish new and further existing life cycle impact assessment categories.

After its termination, efforts were undertaken to transform the outcomes of the project into one working methodology, also called LC-Impact. All categories of LC-Impact are thus based on either existing impact categories from previous approaches, such as ReCiPe[1], or are based on peer-reviewed publications. The complete information and background to each chapter can be found in individual chapters on www.lc-impact.eu. In Table S1 the most relevant background information for each impact category we used here, is given. We assumed the same CF for fossil and biogenic CO_2.

Table S1: Most important background documents for each impact category beyond the LC-Impact[2] chapters themselves.

**Impact category**	**Taxa covered**	**References**
Climate change	Global average	ReCiPe update (2014)[3], Urban (2015)[4], Hanafiah et al. (2011)[5]
Freshwater eutrophication	Freshwater fish	Helmes et al.(2012),[6] Azevedo et al. (2013a,b)[7,8], Azevedo (2014)[9], Scherer et al. (2015)[10]
Marine eutrophication	Actinopterygii, Chondrichthyes, Liliopsida, Anthozoa, Halothuroidea, Gastropoda (occurring in marine neritic habitats)	Cosme et al.(2015,2016)[11,12]
Water consumption	Mammalia, Aves, Amphibia, Reptilia, Tracheophyta	Verones et al.(2013a,b, submitted)[13-15]
Land use	Mammalia, Aves, Amphibia, Reptilia, Tracheophyta	Chaudhary et al. (2015)[16]
Terrestrial acidification	Tracheophyta	Roy et al. (2012a,b,2014)[17-19], Azevedo et al. (2013)[20]

Some of the impacts calculated in the main paper are above a PDF of 1, i.e. well above what one would commonly assume to be a fraction. This is mostly caused through the time-integration of the impacts since we integrate the continuous impact one pulse has over the entire timeframe considered. For example, a pulse of a CO_2 emission will stay active within the atmosphere for years and constantly contributes to climate change. The period considered for climate change is usually 100 years. Impacts from e.g. eutrophication, on the other hand, have a nearly instantaneous effect and cease after the nutrients excess has been absorbed. Nevertheless has each emission or resource use a time dimension associated with it (use per year or emission per year). Therefore, there is also a time dimension included in the characterization factors.

Alignment of production and impact locations

Since trade data is available on a national level only, we aggregated all characterization factors on the corresponding level. However, this raises the question whether production happens actually in the areas where impacts are likely to happen or not. Such an alignment in spatial detail is relevant for example for water consumption. Due to the rather high impacts in the US, we use the US as an example here. We use maize, wheat and cotton as examples, being among the most important crops in the US (Figure S1). Maps for the use of irrigation water for crops are from ref[21].

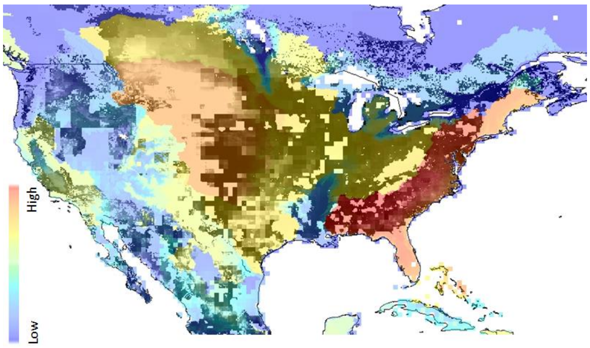

A)

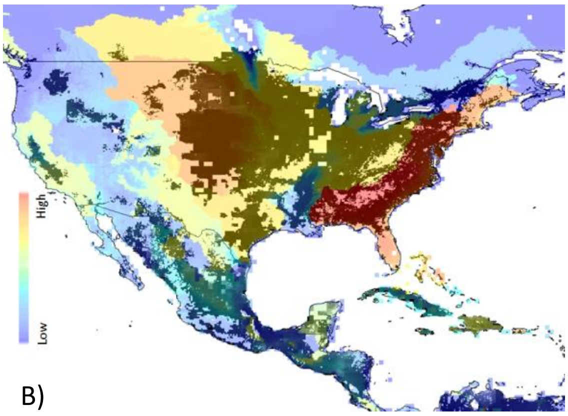

B)

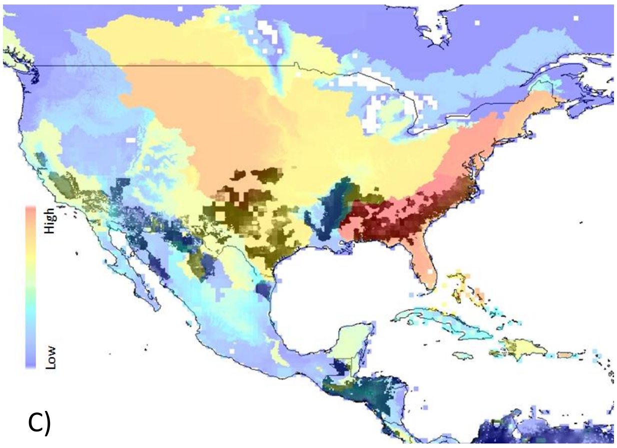

Figure S1: Overlay of the characterization factor for water consumption in the US with the irrigated areas of A) wheat, B) maize and C) cotton. Map created with ArcMap 10.2(ww.esri.com/software/arcgis)[22].

The water for irrigation is mainly used for in areas with a high characterization factor. This contributes to the explanation of the magnitude of the impact in the US.

2. Pressure footprint vs. Impact footprint

In Table S1 we can see the difference between net exporting and importing countries in terms of pressure footprints or impact footprints.

Table S2 –Number of net importer and net exporter countries, in terms of Resource Footprints vs Impact Footprints. For land, water, and air pollution footprints the pattern reverses from a plurality of importers using resources from a subset of exporters, to a plurality of net exporters.

	Pressure Footprint (traditional method)		Impact Footprint (new method)		
Impact category	# of net importers	# of net exporters	# of net importers	# of net exporters	
Climate change (CO2, CH4)	**127**	63	**127**	63	
Climate change (N2O)	**138**	52	**138**	52	
Terr. acidification (NOx)	**145**	45	47	**143**	*Pattern reversed*
Terr. acidification (NH3)	**139**	51	39	**151**	*Pattern reversed*
Terr. acidification (SO2)	**145**	45	39	**151**	*Pattern reversed*
Cropland	**103**	87	46	**144**	*Pattern reversed*
Marine eutrophication	46	144	34	**156**	
Freshwater eutrophication	46	144	45	**145**	
Forest land	**103**	87	29	**161**	*Pattern reversed*
Water consumption	**109**	81	53	**137**	*Pattern reversed*

3. Maps and scatterplots of pressure and impact footprints

In the following figures (Figures S1 to S9), the maps of the impact footprints and the scatterplot showing the comparison between pressure footprints and impact footprints are presented. The later show the standardized relations of the pressure vs. impact footprint relations. Due to many small countries with relatively low impacts/pressures, we also provide log – standardized graphs which better depict the relationships for these small countries (Figures S11–S21).

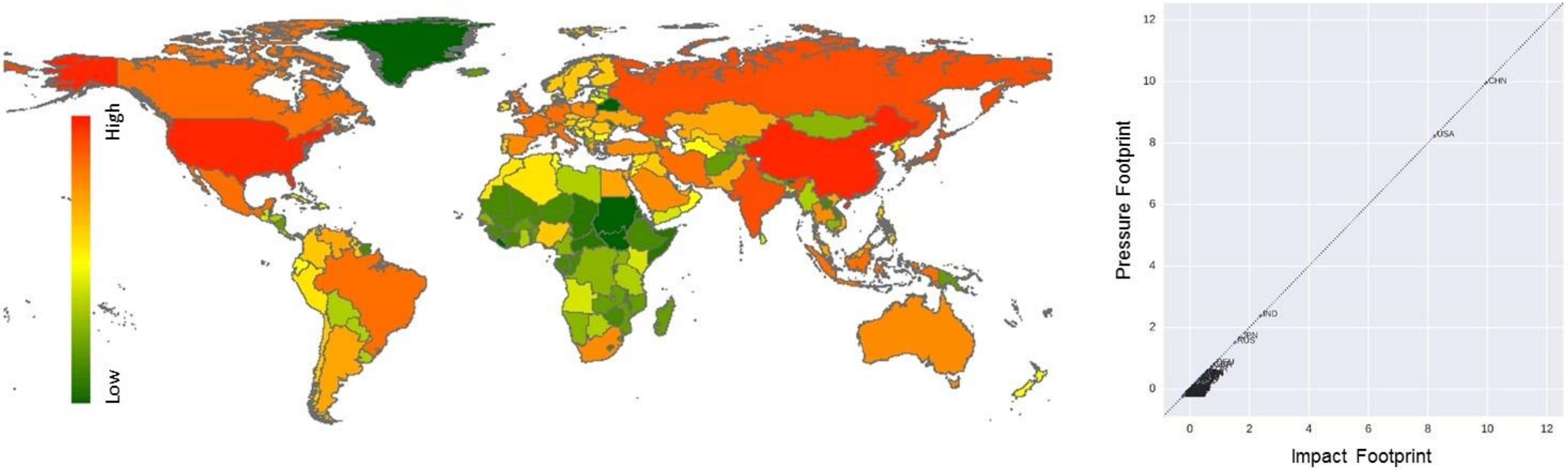

Figure S2: The impact footprint of nations for CO_2 emissions. In the scatterplot the footprint of nations can differ sharply when viewed as traditional pressure footprints (y-axis) vs. as impact footprints (x-axis)(both axis standardized (see methods), dotted line representing similar pressure and impact footprints). Since factors for CO_2 are global in LC-Impact, pressure and impact footprints are not differing. Map created with ArcMap 10.2(ww.esri.com/software/arcgis)[22].

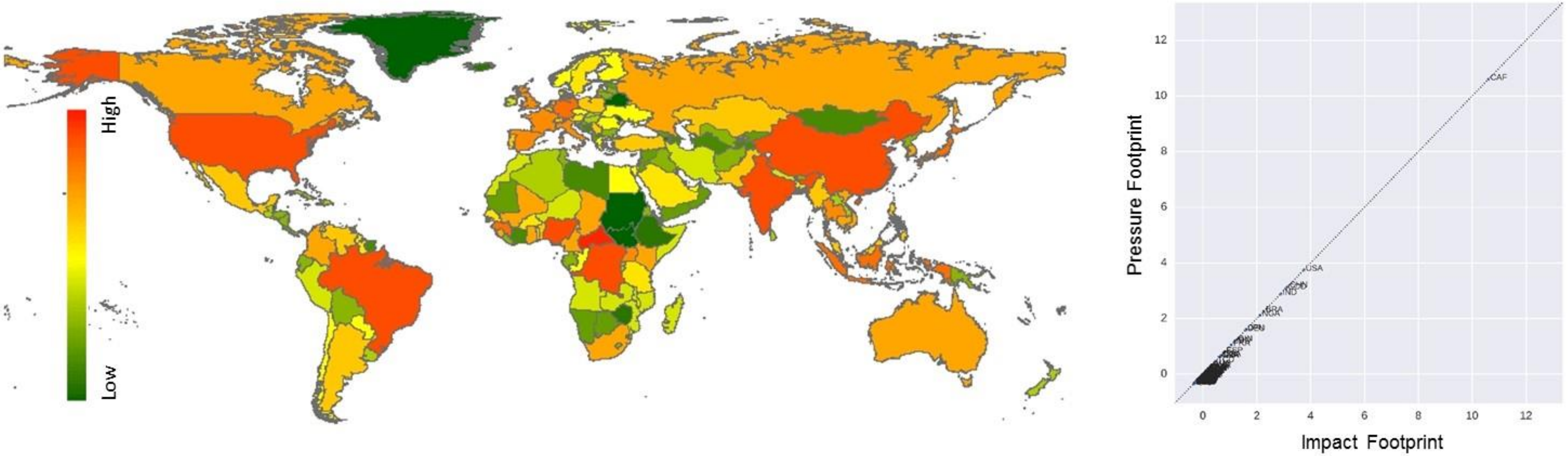

Figure S3: The impact footprint of nations for biogenic CO_2 emissions. In the scatterplot the footprint of nations can differ sharply when viewed as traditional pressure footprints (y-axis) vs. as impact footprints (x-axis)(both axis standardized (see methods), dotted line representing similar pressure and impact footprints). Since factors for biogenic CO_2 are global in LC-Impact, pressure and impact footprints are not differing. Map created with ArcMap 10.2(ww.esri.com/software/arcgis)[22].

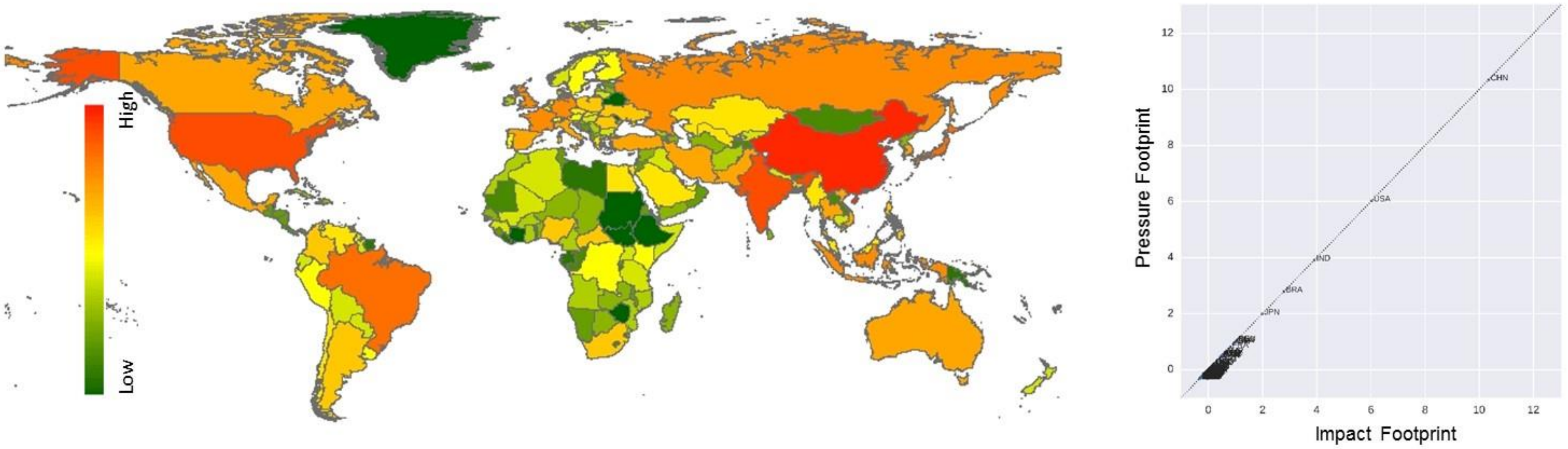

Figure S4: The impact footprint of nations for CH_4 emissions. In the scatterplot the footprint of nations can differ sharply when viewed as traditional pressure footprints (y-axis) vs. as impact footprints (x-axis)(both axis standardized (see methods), dotted line representing similar pressure and impact footprints). Since factors for CH_4 are global in LC-Impact, pressure and impact footprints are not differing. Map created with ArcMap 10.2(ww.esri.com/software/arcgis)[22].

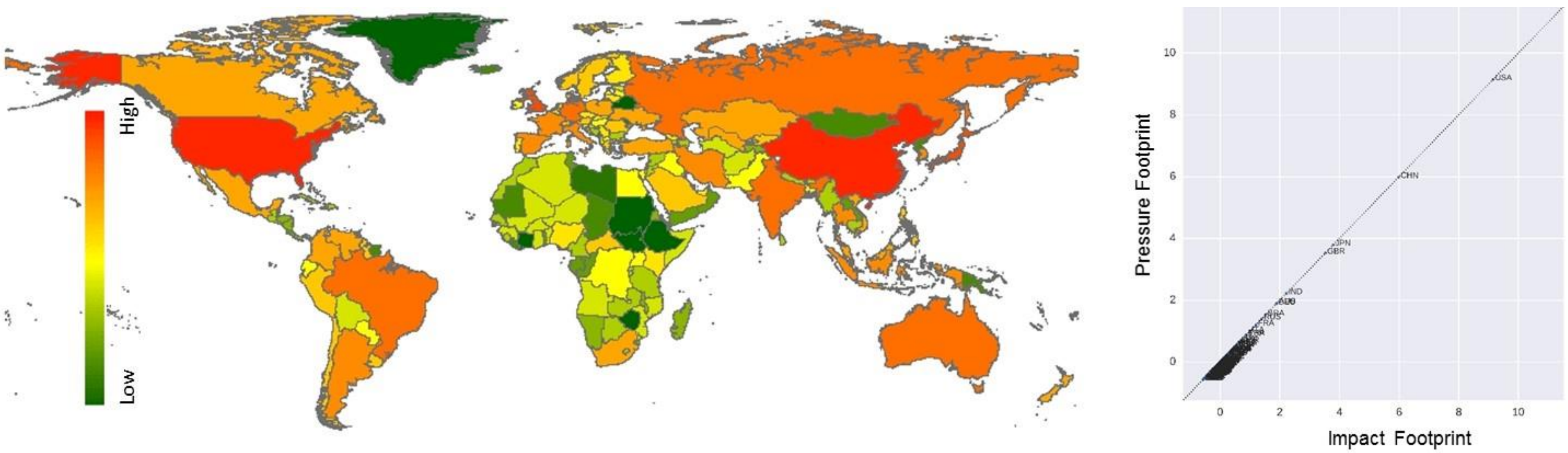

Figure S5: The impact footprint of nations for N_2O emissions. In the scatterplot the footprint of nations can differ sharply when viewed as traditional pressure footprints (y-axis) vs. as impact footprints (x-axis)(both axis standardized (see methods), dotted line representing similar pressure and impact footprints). Since factors for N_2O are global in LC-Impact, pressure and impact footprints are not differing. Map created with ArcMap 10.2(ww.esri.com/software/arcgis)[22].

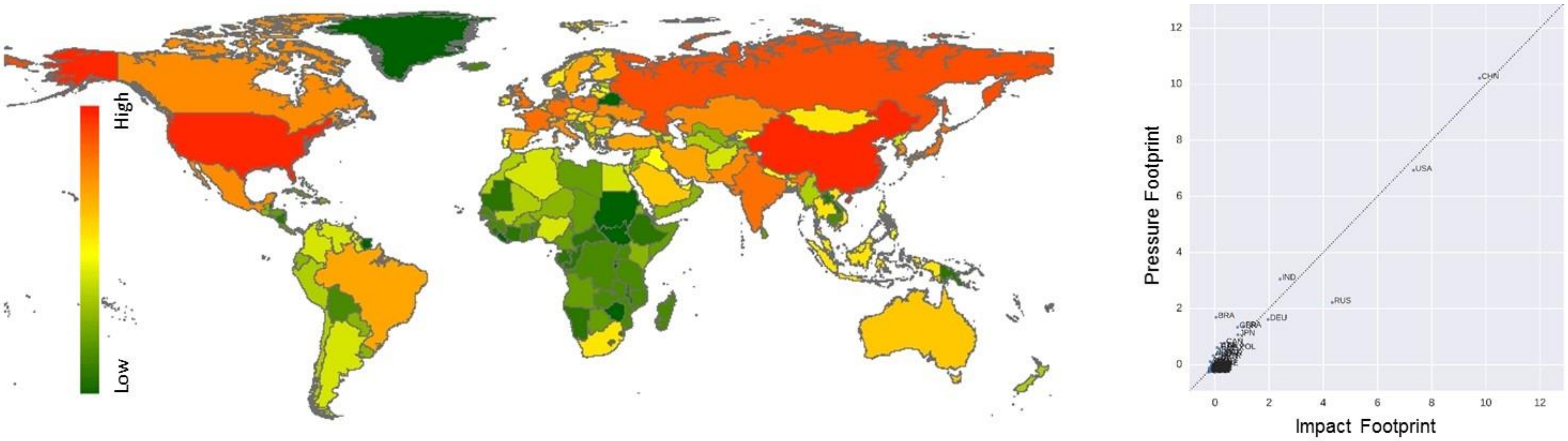

Figure S6: The impact footprint of nations for nitrogen emissions, leading to marine eutrophication. In the scatterplot the footprint of nations can differ sharply when viewed as traditional pressure footprints (y-axis) vs. as impact footprints (x-axis)(both axis standardized (see methods), dotted line representing similar pressure and impact footprints). Map created with ArcMap 10.2(ww.esri.com/software/arcgis)[22].

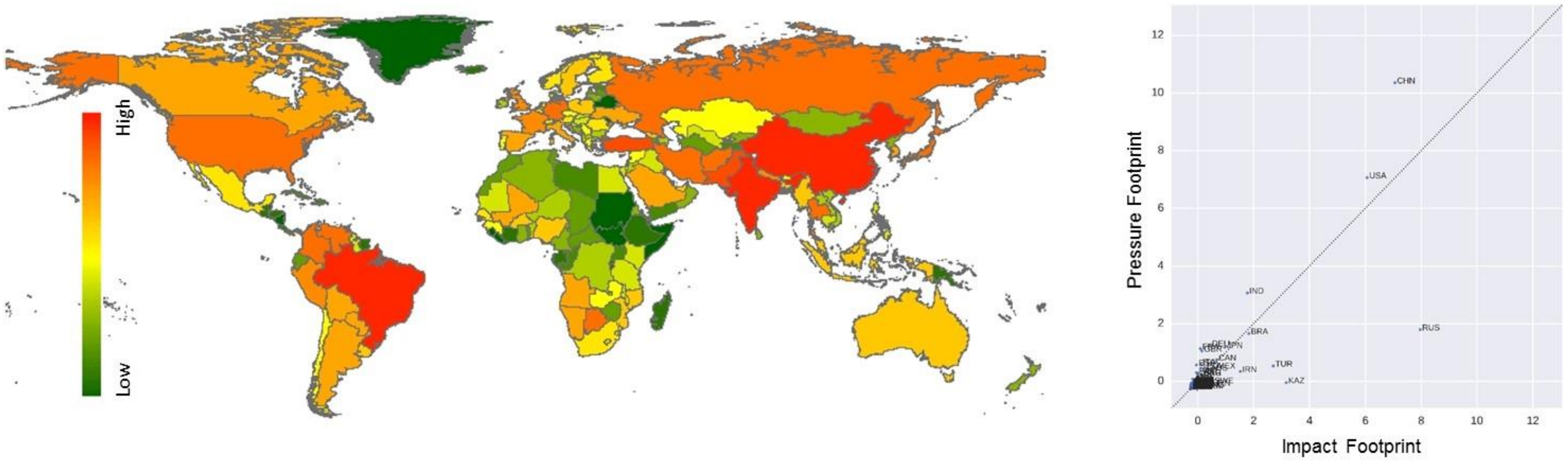

Figure S7: The impact footprint of nations for phosphorus emissions, leading to freshwater eutrophication. In the scatterplot the footprint of nations can differ sharply when viewed as traditional pressure footprints (y-axis) vs. as impact footprints (x-axis)(both axis standardized (see methods), dotted line representing similar pressure and impact footprints). Map created with ArcMap 10.2(ww.esri.com/software/arcgis)[22]

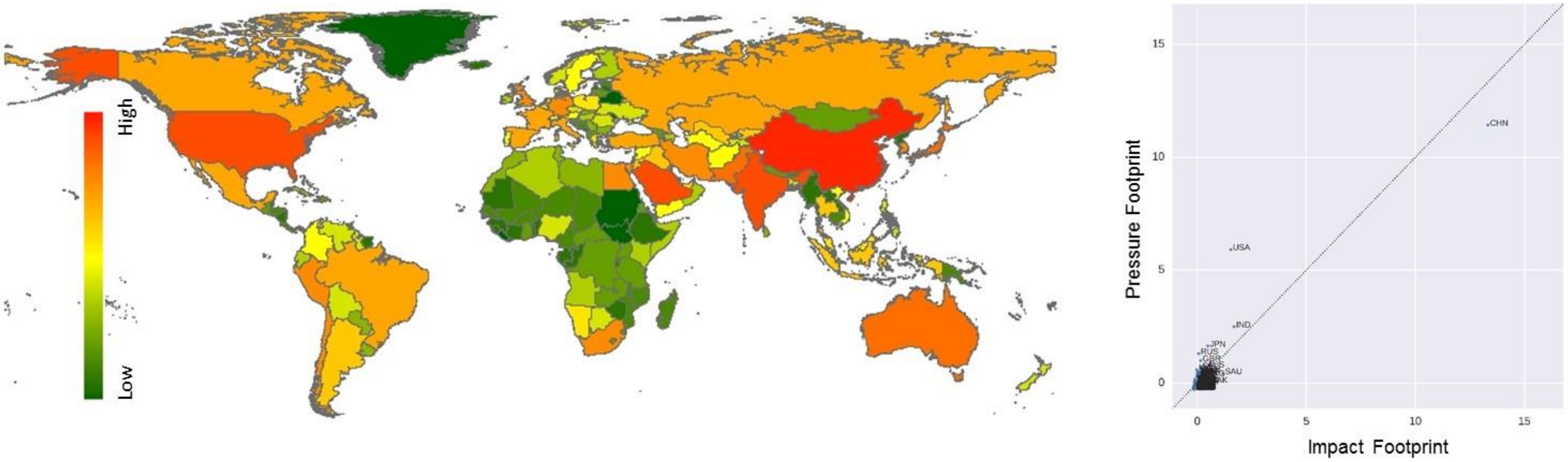

Figure S8: The impact footprint of nations for SO_2 emissions, leading to terrestrial acidification. In the scatterplot the footprint of nations can differ sharply when viewed as traditional pressure footprints (y-axis) vs. as impact footprints (x-axis)(both axis standardized (see methods), dotted line representing similar pressure and impact footprints). Map created with ArcMap 10.2(ww.esri.com/software/arcgis)[22].

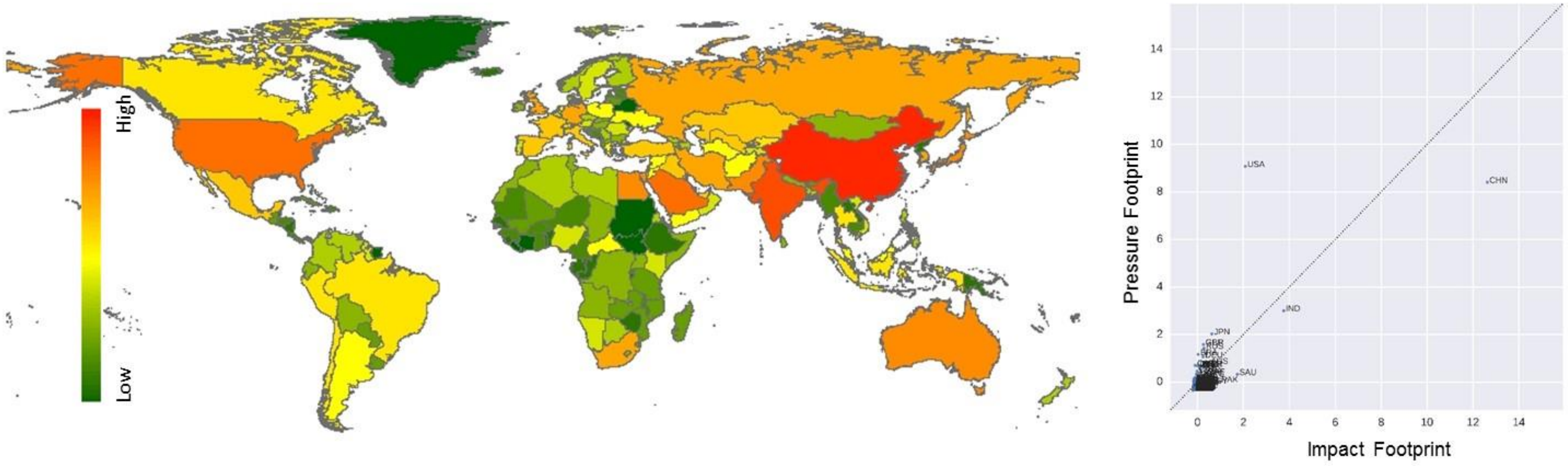

Figure S9: The impact footprint of nations for NO_x emissions, leading to terrestrial acidification. In the scatterplot the footprint of nations can differ sharply when viewed as traditional pressure footprints (y-axis) vs. as impact footprints (x-axis)(both axis standardized (see methods), dotted line representing similar pressure and impact footprints). Map created with ArcMap 10.2(ww.esri.com/software/arcgis)[22].

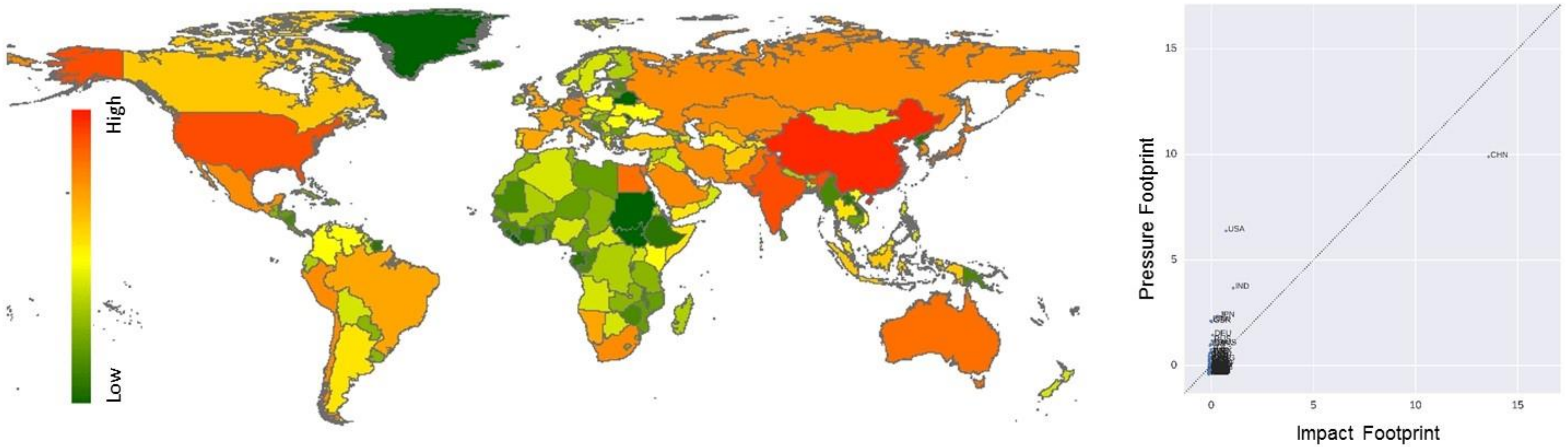

Figure S10: The impact footprint of nations for NH_3 emissions, leading to terrestrial acidification. In the scatterplot the footprint of nations can differ sharply when viewed as traditional pressure footprints (y-axis) vs. as impact footprints (x-axis)(both axis standardized (see methods), dotted line representing similar pressure and impact footprints). Map created with ArcMap 10.2(ww.esri.com/software/arcgis)[22].

Figures S11-S21 depict the standardized Pressure vs Impact footprints of all dicscussed accounts (corresponding to Figure 3 of the main text and Figures S2 – S11 in the Supplements) on a double logarithmic scale. This scale provides a better representation of the footprints relationships for countries with smaller footprints. In order to remove negative values for the logarthmic transformation, the minimal occuring standardised value (per account) was added to all standardized values of the corresponding account.

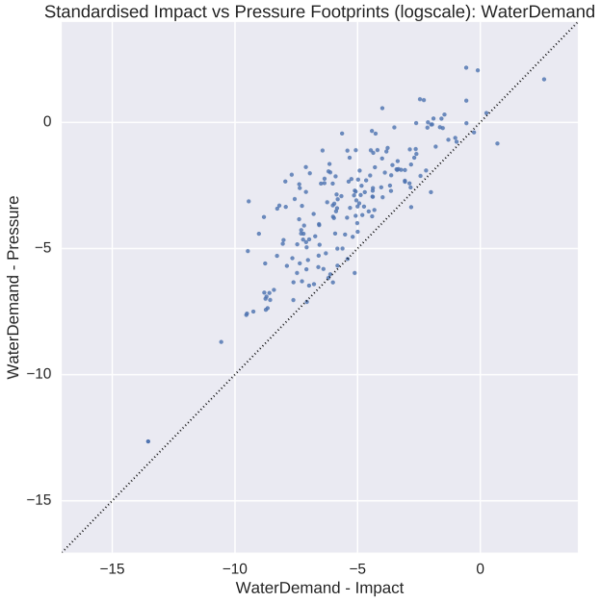

Fig S11: Standardized Pressure vs Impact Footprints for Water Demand with logarithmic x and y axis. For the majority of countries footprints differ substantially when viewed as traditional pressure footprints (y-axis) vs. as impact footprints (x-axis).

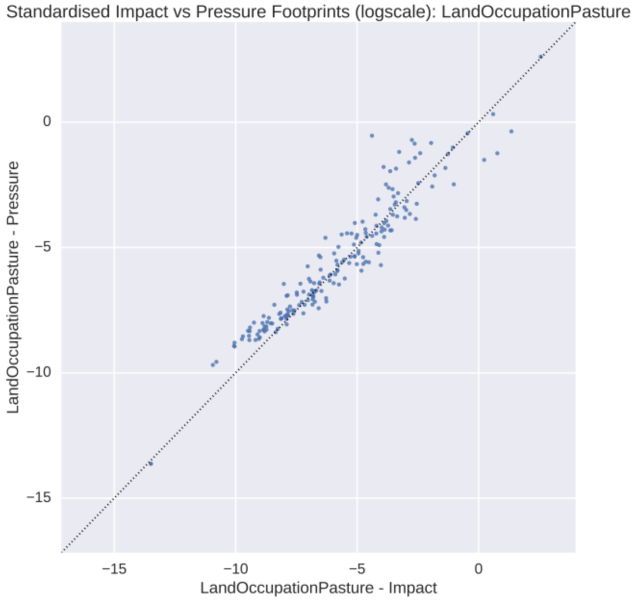

Fig S12: Standardized Pressure vs Impact Footprints for pasture related land occupation with logarithmic x and y axis. For the majority of countries footprints differ substantially when viewed as traditional pressure footprints vs. as impact footprints.

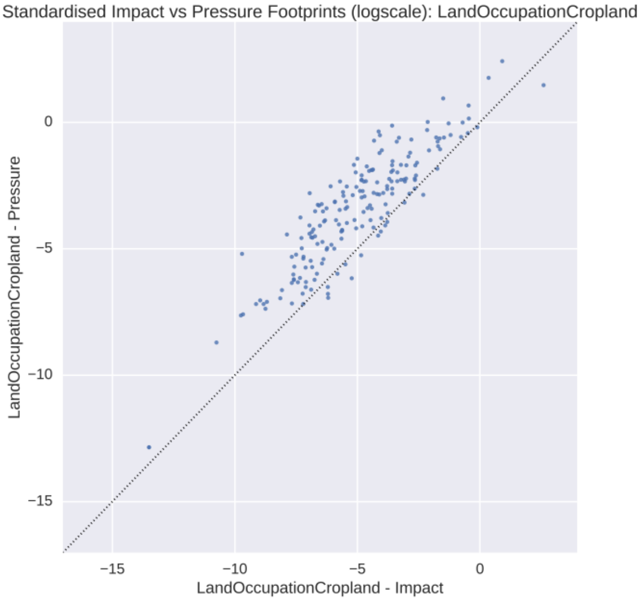

Fig S13: Standardized Pressure vs Impact Footprints for cropland related land occupation with logarithmic x and y axis. For the majority of countries footprints differ substantially when viewed as traditional pressure footprints (y-axis) vs. as impact footprints (x-axis).

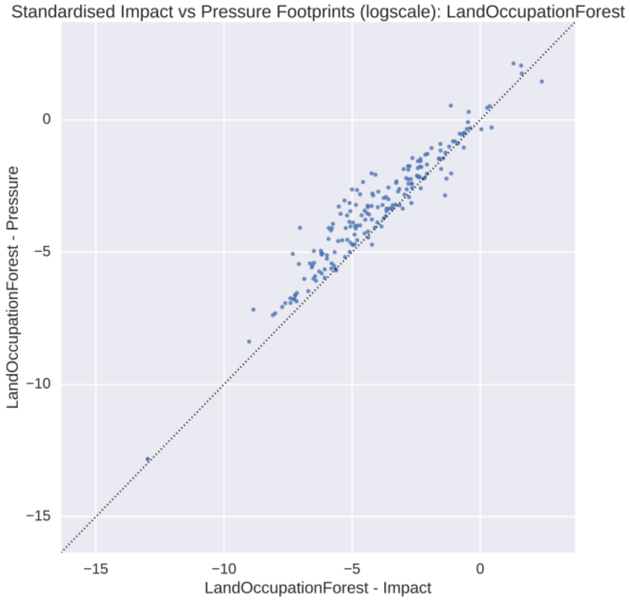

Fig S13: Standardized Pressure vs Impact Footprints for forest related land occupation with logarithmic x and y axis. For the majority of countries footprints differ substantially when viewed as traditional pressure footprints (y-axis) vs. as impact footprints (x-axis).

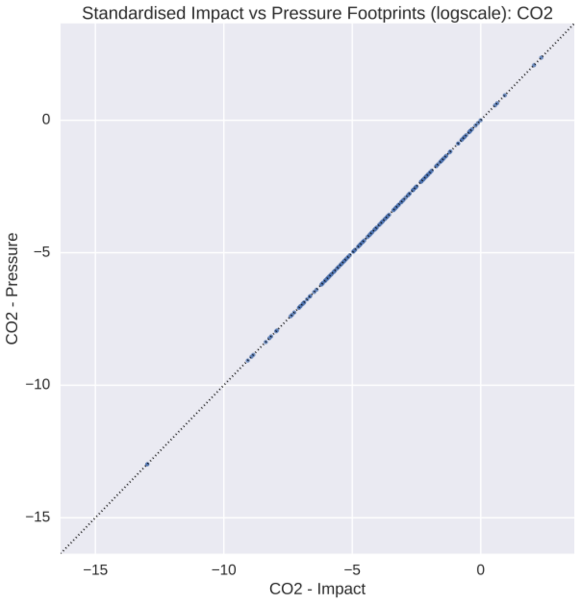

Fig S13: Standardized Pressure vs Impact Footprints for CO_2 emissions with logarithmic x and y axis. Due to global impact characterization factors for CO_2, the standardized footprints of nations for pressure and impact footprints are equal – all countries lie on the dotted line representing similar footprints.

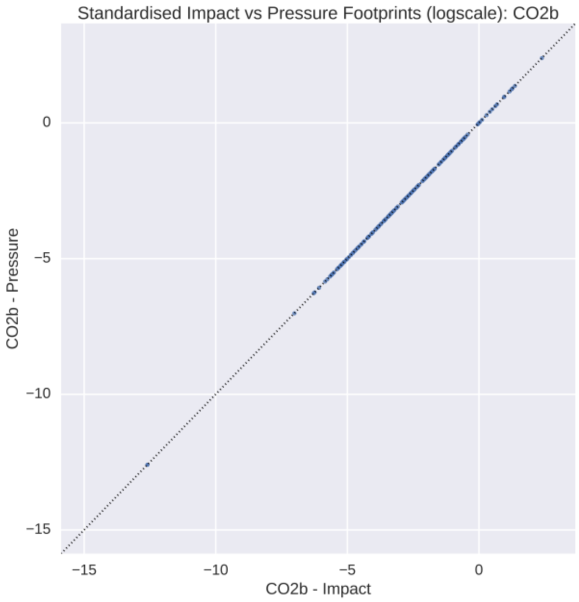

Fig S14: Standardized Pressure vs Impact Footprints for biogenic CO_2 emissions with logarithmic x and y axis. Due to global impact characterization factors for biogenic CO_2, the standardized footprints of nations for pressure and impact footprints are equal – all countries lie on the dotted line representing similar footprints.

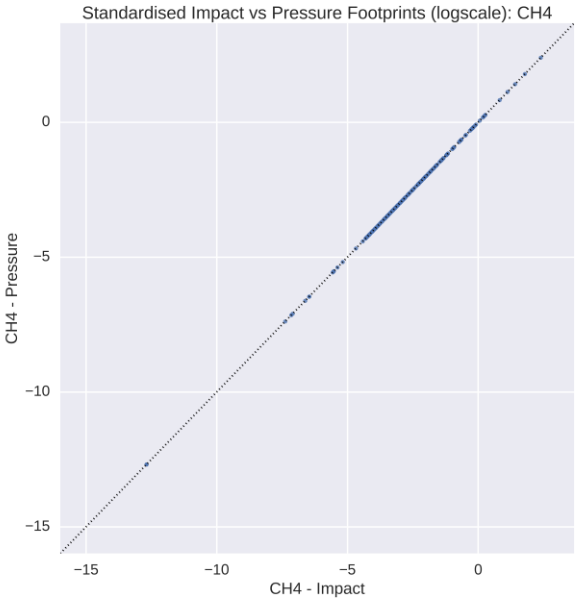

Fig S15: Standardized Pressure vs Impact Footprints for CH_4 emissions with logarithmic x and y axis. Due to global impact characterization factors for CH_4, the standardized footprints of nations for pressure and impact footprints are equal – all countries lie on the dotted line representing similar footprints.

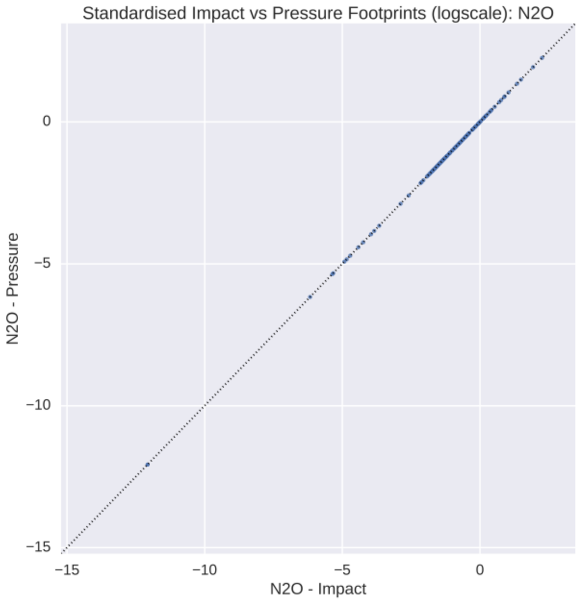

Fig S16: Standardized Pressure vs Impact Footprints for N_2O emissions with logarithmic x and y axis. Due to global impact characterization factors for N_2O, the standardized footprints of nations for pressure and impact footprints are equal – all countries lie on the dotted line representing similar footprints.

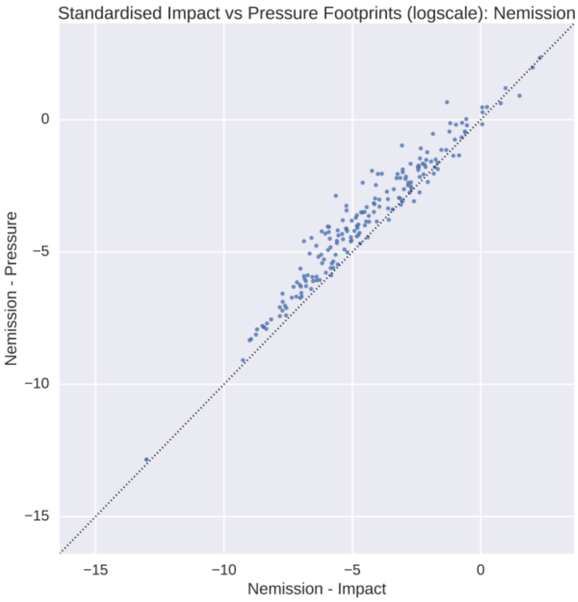

Fig S17: Standardized Pressure vs Impact Footprints for Nitrogen emissions related to Eutrophication with logarithmic x and y axis. For the majority of countries footprints differ substantially when viewed as traditional pressure footprints (y-axis) vs. as impact footprints (x-axis).

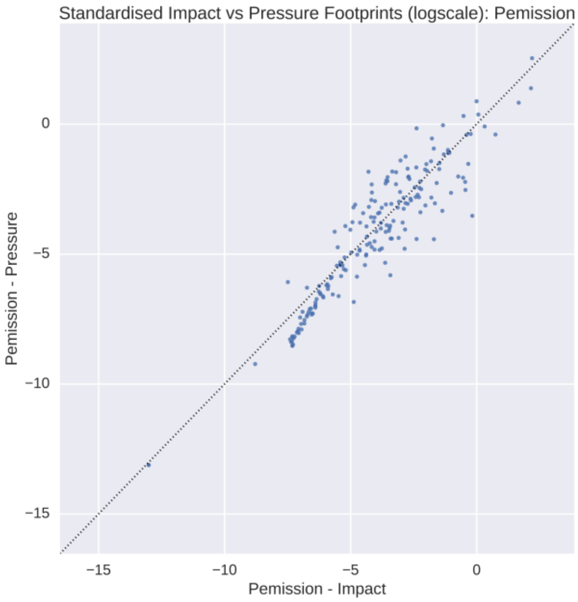

Fig S18: Standardized Pressure vs Impact Footprints for Phosphorus emissions related to Eutrophication with logarithmic x and y axis. For the majority of countries footprints differ substantially when viewed as traditional pressure footprints (y-axis) vs. as impact footprints (x-axis).

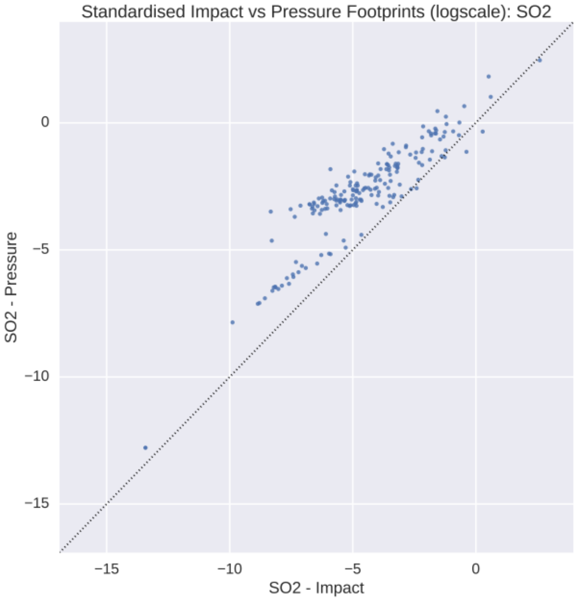

Fig S19: Standardized Pressure vs Impact Footprints SO_2 with logarithmic x and y axis. For the majority of countries footprints differ substantially when viewed as traditional pressure footprints (y-axis) vs. as impact footprints (x-axis).

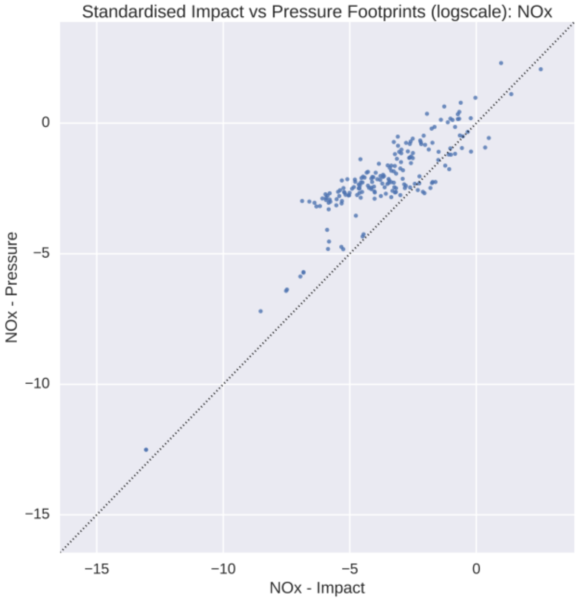

Fig S20: Standardized Pressure vs Impact Footprints NO_x with logarithmic x and y axis. For the majority of countries footprints differ substantially when viewed as traditional pressure footprints (y-axis) vs. as impact footprints (x-axis).

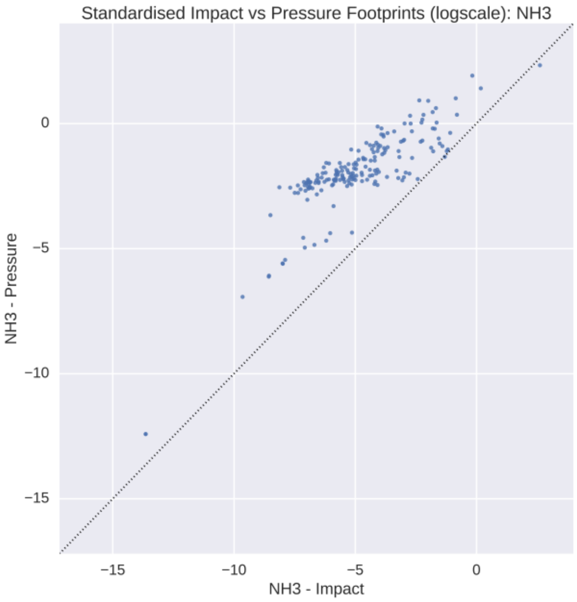

Fig S21: Standardized Pressure vs Impact Footprints NH_3 with logarithmic x and y axis. For the majority of countries footprints differ substantially when viewed as traditional pressure footprints (y-axis) vs. as impact footprints (x-axis).

4. Detailed impact footprints and resource footprints per country

See Excel and bilateral trade results files for download on the journal webpage. Bilateral trade results (embodied flows from each country to each other country) are measured both as Impact Footprints and as Pressure Footprints.

5. References

1 Goedkoop, M. *et al.* ReCiPe 2008: A life cycle impact assessment method which comprises harmonised category indicators at the midpoint and endpoint levels. First edition. Report i: Characterization. (Ruimte en Milieu, Ministerie van Volkshuisvesting, Ruimtelijke Ordening en Milieubeheer, The Netherlands, 2009).

2 Verones, F. *et al. LC-IMPACT Version 0.5- A spatially differentiated life cycle impact assessment approach* [last accessed 19 July 2016]; Available from:<http://www.lc-impact.eu/>.

3 Huijbregts, M. A. J., Steinmann, Z. J. N., Stam, G. & Van Zelm, R. Update of emission-related impact categories in ReCiPe2008. (Department of Environmental Science, Radboud University Nijmegen, 2014).

4 Urban, M. C. Accelerating extinction risk from climate change. *Science* **348**, 571-573 (2015).

5 Hanafiah, M. M., Xenopoulos, M. A., Pfister, S., Leuven, R. S. & Huijbregts, M. A. J. Characterization Factors for Water Consumption and Greenhouse Gas Emissions Based on Freshwater Fish Species Extinction. *Environ. Sci. Technol.* **45**, 5572-5278 (2011).

6 Helmes, R. J. K., Huijbregts, M. A. J., Henderson, A. D. & Jolliet, O. Spatially explicit fate factors of phosphorous emissions to freshwater at the global scale. *Int J LCA* **17**, 646-654, doi:10.1007/s11367-012-0382-2 (2012).

7 Azevedo, L. B., Henderson, A. D., Van Zelm, R., Jolliet, O. & Huijbregts, M. A. J. Assessing the Importance of Spatial Variability versus Model Choices in Life Cycle Impact Assessment: The Case of Freshwater Eutrophication in Europe. *Environmental Science and Technology* **47**, 13565-13570 (2013).

8 Azevedo, L. B. *et al.* Species richness - phosphorus relationships for lakes and streams worldwide. *Global Ecology and Biogeography* **22**, 1304-1314 (2013).

9 Azevedo, L. B. Development and application of stressor - response relationships of nutrients. Chapter 8. Ph.D. thesis, Radboud University of Nijmegen, The Netherlands. http://repository.ubn.ru.nl/handle/2066/129667. (2014).

10 Scherer, L. & Pfister, S. Modelling spatially explicit impacts from phosphorus emissions in agriculture. *Int J LCA* **20**, 785-795, doi:10.1007/s11367-015-0880-0 (2015).

11 Cosme, N. & Hauschild, M. Z. Effect Factors for marine eutrophication in LCIA based on species sensitivity to hypoxia. *Ecological Indicators* **69**, 453-462 (2016).

12 Cosme, N., Koski, M. & Hauschild, M. Z. Exposure factors for marine eutrophication impacts assessment based on a mechanistic biological model. *Ecological Modelling* **317**, 50-63, doi:http://dx.doi.org/10.1016/j.ecolmodel.2015.09.005 (2015).

13 Verones, F., Pfister, S. & Hellweg, S. Quantifying area changes of internationally important wetlands due to water consumption in LCA. *Environ. Sci. Technol.* **47**, 9799-9807 (2013).

14 Verones, F., Pfister, S., van Zelm, R. & Hellweg, S. Biodiversity impacts from water consumption on a global scale for use in life cycle assessment. (submitted).

15 Verones, F. *et al.* Effects of consumptive water use on wetlands of international importance. *Environ. Sci. Technol.* **47**, 12248-12257 (2013).

16 Chaudhary, A., Verones, F., De Baan, L. & Hellweg, S. Quantifying Land Use Impacts on Biodiversity: Combining Species-Area Models and Vulnerability Indicators. *Environ. Sci. Technol.* **49**, 9987-9995 (2015).

17 Roy, P.-O. *et al.* Characterization factors for terrestrial acidification at the global scale: A systematic analysis of spatial variability and uncertainty. *Science of The Total Environment* **500–501**, 270-276 (2014).

18 Roy, P.-O., Deschênes, L. & Margni, M. Life Cycle Impact Assessment of Terrestrial Acidification: Modeling Spatially Explicit Soil Sensitivity at the Global Scale. *Environmental Science & Technology* **46**, 8270-8278, doi:10.1021/es3013563 (2012).

19 Roy, P.-O., Huijbregts, M., Deschênes, L. & Margni, M. Spatially-differentiated atmospheric source–receptor relationships for nitrogen oxides, sulfur oxides and ammonia emissions at the global scale for life cycle impact assessment. *Atmospheric Environment* **62**, 74-81, doi:http://dx.doi.org/10.1016/j.atmosenv.2012.07.069 (2012).

20 Azevedo, L. B., Van Zelm, R., Hendriks, A. J., Bobbink, R. & Huijbregts, M. A. J. Global assessments of the effects of terrestrial acidification on plant species richness. *Environmental Pollution* **174**, 10-15 (2013).

21 Pfister, S., Bayer, P., Koehler, A. & Hellweg, S. Environmental Impacts of Water Use in Global Crop Production: Hotspots and Trade-Offs with Land Use. *Environ. Sci. Technol.* **45**, 5761-5768 (2011).

22 ArcGIS Desktop10.2. http://www.esri.com/software/arcgis (2013).
